# Contrasting Host-Parasite Population Structure: Morphology and Mitogenomics of a Parasitic Flatworm on Pelagic Deepwater Cichlid Fishes from Lake Tanganyika

**DOI:** 10.3390/biology10080797

**Published:** 2021-08-18

**Authors:** Nikol Kmentová, Christoph Hahn, Stephan Koblmüller, Holger Zimmermann, Jiří Vorel, Tom Artois, Milan Gelnar, Maarten P. M. Vanhove

**Affiliations:** 1 Department of Botany and Zoology, Faculty of Science, Masaryk University, Kotlářská 2, 611 37 Brno, Czech Republic; vorel@mail.muni.cz (J.V.); gelnar@sci.muni.cz (M.G.); maarten.vanhove@uhasselt.be (M.P.M.V.); 2 Research Group Zoology: Biodiversity & Toxicology, Centre for Environmental Sciences, Hasselt University, Agoralaan Gebouw D, B-3590 Diepenbeek, Belgium; tom.artois@uhasselt.be; 3 Institute of Biology, University of Graz, Universitätsplatz 2, A-8010 Graz, Austria; christoph.hahn@uni-graz.at (C.H.); stephan.koblmueller@uni-graz.at (S.K.); holger.zimmermann@uni-graz.at (H.Z.); 4 The Czech Academy of Sciences, Institute of Vertebrate Biology, Květná 8, 603 65 Brno, Czech Republic

**Keywords:** monogenea, *Cichlidogyrus*, Bathybatini, *cox*1, PoolSeq

## Abstract

**Simple Summary:**

Species richness in open water areas is generally lower than in coastal zones. Fish parasites have been targeted as biological tags potentially magnifying biological patterns of their hosts including long-distance migrations notoriously difficult to trace. Lake Tanganyika (Africa) is an ideal place to study general mechanisms of host-parasite interactions in an open water environment and *Cichlidogyrus*, a monogenean flatworm lineage also present in Lake Tanganyika, has been proposed as a model system to study parasite-host relationships. The present study revealed the lake-wide occurrence (600 km) of *Cichlidogyrus casuarinus*, a parasite with a broad host range infecting pelagic fishes endemic to Lake Tanganyika. Our comparative approach highlighted incongruence between morphological and genetic differentiation of the populations of *Cichlidogyrus casuarinus*. Our results show a limitation of the parasite’s magnifying potential for the focal host species due to the parasites’ broad host range including highly mobile host species. Using different sequencing technologies, the study further provides the first assessment of the genetic variation of mitochondrial data in *Cichlidogyrus* showing contrasting patterns within and between parasite species. Given the now considerable baseline knowledge on its morphological and genetic variation, we propose *C. casuarinus* as a model to study (1) mechanisms driving host range and (2) the role of phenotypic plasticity in diversification and speciation.

**Abstract:**

Little phylogeographic structure is presumed for highly mobile species in pelagic zones. Lake Tanganyika is a unique ecosystem with a speciose and largely endemic fauna famous for its remarkable evolutionary history. In bathybatine cichlid fishes, the pattern of lake-wide population differentiation differs among species. We assessed the congruence between the phylogeographic structure of bathybatine cichlids and their parasitic flatworm *Cichlidogyrus casuarinus* to test the magnifying glass hypothesis. Additionally, we evaluated the use of a PoolSeq approach to study intraspecific variation in dactylogyrid monogeneans. The lake-wide population structure of *C. casuarinus* ex *Hemibates stenosoma* was assessed based on a portion of the *cox*1 gene combined with morphological characterisation. Additionally, intraspecific mitogenomic variation among 80 parasite samples from one spatially constrained metapopulation was assessed using shotgun NGS. While no clear geographic genetic structure was detected in parasites, both geographic and host-related phenotypic variation was apparent. The incongruence with the genetic north-south gradient observed in *H. stenosoma* may be explained by the broad host range of this flatworm including eupelagic bathybatine host species that form panmictic populations across the lake. In addition, we present the first parasite mitogenome from Lake Tanganyika and propose a methodological framework for studying the intraspecific mitogenomic variation of dactylogyrid monogeneans.

## 1. Introduction

Species richness in the pelagic zones of large water bodies is generally low compared to littoral habitats. This is not only true for marine ecosystems [1], but also large lakes [2,3]. The often uniform appearance of highly mobile pelagic species, such as fish, reflects the lack of physical barriers to gene flow and of resource-based diversification [4] related to trophic relationships [4,5]. Nevertheless, in many cases it remains notoriously difficult to infer gene flow across the open water and consequently, to draw a connection between panmixia and the specialisation in open water habitats [6,7].

Similar to the rather uniform fish host species composition in pelagic habitats, low parasite species diversity has been observed in the open water and deep sea ecosystems worldwide [8,9,10,11,12,13,14]. Due to their shorter generation time and high mutation rate, parasite lineages are often more species-rich than their hosts, and accelerated microevolution is also visible in their population structure [15]. Therefore, distribution patterns of parasites have been suggested to mirror and further magnify population structure, migration patterns and historical distribution of their hosts [16,17,18,19]. However, evolutionary mechanisms in most parasite taxa remain poorly studied, especially at the population level. Due to the diversity of life strategies and host taxa involved, flatworms earned the label “masters of parasitism” [20]. Monogenean parasites, a group of neodermatan flatworms, are monoxenous (individual life cycles depend on single host individuals) and often display high levels of host-specificity [21]. They are, therefore, considered a prime candidate model for using parasites as tags for their hosts’ history and ecology.

Unlike the open sea, pelagic zones of lakes are geographically confined and easier to monitor as a whole. Therefore, they could serve as more approachable systems for studying evolutionary processes and host–parasite relationships among open water taxa. Lake Tanganyika is well suited to study parasite distribution patterns because it is highly isolated from surrounding water bodies and is home to a species-rich endemic cichlid species assemblage, a famous textbook model of evolution in natural conditions [22,23,24,25], infected by dactylogyrid monogeneans belonging to *Cichlidogyrus* Paperna, 1960 (Monopisthocotylea, Dactylogyridae). Like their hosts, these monogeneans comprise a stunning species diversity in Lake Tanganyika (and elsewhere) [26,27]. Therefore, *Cichlidogyrus* was recently suggested as a model genus for studying host-parasite interactions [27]. Lake Tanganyika comprises a large but still monitorable pelagic zone with layer stratification (epi-, meso-, and bathypelagic) inhabited by schooling freshwater species of sardines (Clupeiformes, Clupeidae), their latid predators (Perciformes, Latidae) as well as bentho- and eupelagic endemic cichlid lineages (Cichliformes, Cichlidae) belonging to the tribes Bathybatini, Boulengerochromini, Benthochromini, Perrisodini and Trematocarini [28,29]. Decreased levels of parasite species richness are often connected with low host-specificity, usually described as the number of different host species a certain parasite species infects [30]. So far, various levels of host-specificity in monogeneans infecting cichlid fishes have been recorded in the lake [31,32]. Mechanisms causing this variation in host-specificity of parasites still remain largely unknown in natural parasite–host systems [27]. The combination of high host dispersal capacities and low host population densities was proposed to cause reduced parasite host-specificity in deep waters [33,34,35], while the former has been suggested to affect the morphology of monogenean populations in Lake Tanganyika [36,37]. This is likely also the case for *Cichlidogyrus casuarinus* Pariselle, Muterezi Bukinga & Vanhove, 2015, which was classified as an intermediate generalist (infecting host species from more than one genus, following [38]) infecting bentho- and eupelagic bathybatine cichlids in the lake. Discovered about a decade ago [39], the relatively large size of *C. casuarinus* compared to all currently known congeners in Lake Tanganyika facilitates a thorough microscopical investigation [40], and because of its relatively broad host range, it was the first African monogenean to be analysed at a population genetic level [31].

Recent developments in Next-generation Sequencing (NGS) now allow for cost-effective studies of population structure and distribution patterns of aquatic migratory species [41,42] based on genomic data. However, the use of NGS approaches in monogeneans has so far been hindered by their small size and low yields from DNA extraction. The use of whole genome data in parasitology has been restricted to few, mostly model parasite taxa of medical importance such as the agents of malaria [43] and schistosomiasis [44]. Population genomics on monogeneans, sourced from the wild without experimental procedures, is to our knowledge an uncovered field.

Recently, a comparative phylogeographic study on bathybatine cichlids showed that benthopelagic species do display geographic population structure, whereas eupelagic species do not [45]. As no host-related (meta)population structure was found within *C. casuarinus* in the northern basin of Lake Tanganyika [31], we question its magnifying potential on a lake-wide scale. In this study, we investigate the geographic population structure of this parasite. We present a rare comparison of a traditional approach used to study intraspecific diversification in monogeneans, namely morphological characterisation and a classic mitochondrial marker, with an approach using NGS data of the same parasite population. We hypothesize that the parasite’s phylogeographic structure is shaped by the dispersal capacity of the most mobile hosts. Alternatively, isolation by distance would suggest philopatry of *C. casuarinus*.

## 2. Material and Methods

### 2.1. Sampling

Monogenean specimens were isolated from the gills of *Hemibates stenosoma* (Boulenger, 1901) (*n* = 8), and a single individual of *Bathybates graueri* (Steindachner, 1911) purchased from local fishermen in Mpulungu, at the southern tip of Lake Tanganyika, Zambia, in September 2018 and 2019, respectively. Fish specimens were identified to the species level in situ and preserved in 99% EtOH prior to examination. Monogenean individuals were extracted from the gills following the standard protocol [46] and cut into three parts with the attachment organ (haptor) and male copulatory organ (MCO) fixed on slides using Hoyer’s medium and the rest of the tissue was kept apart for molecular characterisation. Species-level confirmation of collected monogenean specimens was carried out based on the original description of *C. casuarianus* [39] with the focus on the morphological details of haptoral structures (mainly length of dorsal bar and first pair of marginal hooks) and MCO. All the collected monogenean specimens were identified as *C. casuarinus.* Parasite voucher material was deposited in the collection of Hasselt University under accession numbers XIV.1.16–1.50 and XIV.2.01–2.33. New data was used in conjunction with previously published (geo-)morphometric and genetic data of *C. casuarinus* from the northern basin [31] to elucidate lake-wide geographical patterns in this monogenean species.

### 2.2. Morphology

#### 2.2.1. Morphometrics

**Figure 1:**
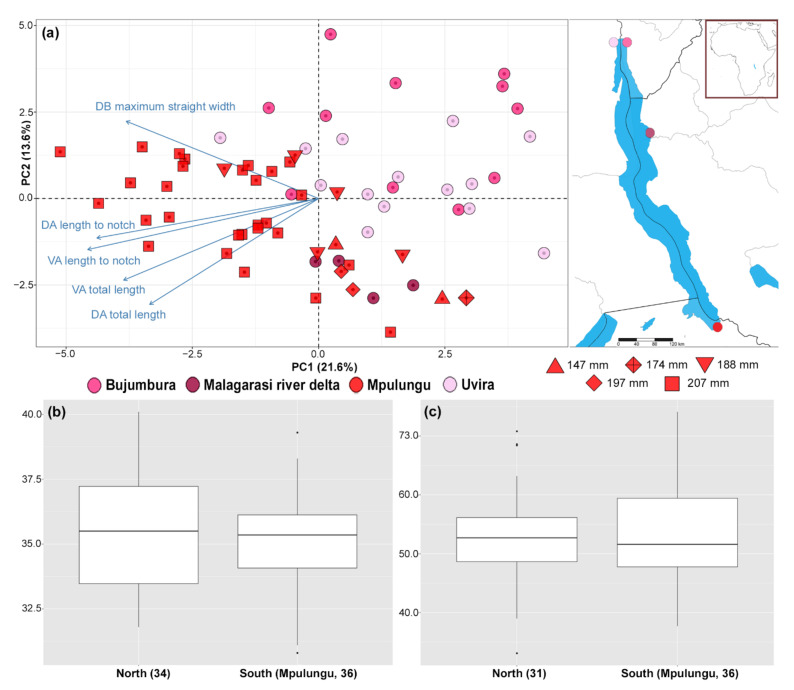
Morphological variation of *Cichlidogyrus casuarinus* ex *Hemibates stenosoma* from Lake Tanganyika collected at several sampling sites based on linear measurements of the haptoral and MCO sclerotised structures. (**a**) A biplot of PCA (first two axes) based on measurements of the haptoral sclerotised structures with the five most contributing variables indicated by arrows. The colours denote the locality of origin combined with signs indicating the size of the fish host that the parasite was extracted from. (**b**) Box-plot graph with a copulatory tube length of *C. casuarinus* (*y*-axis in µm) separated by basin. (**c**) Box-plot graph with a heel length of *C. casuarinus* (*y*-axis in µm) separated by basin. The number of specimens measured is indicated in parentheses.

Detailed characterisation of parasite sclerotised structures was performed on 43 monogenean individuals ex *H. stenosoma* and five ex *B. graueri* (see Table 1 and Table S1). In total, 22 parameters from the haptor and three from the MCO were measured following the terminology of [47]. Given that [31] observed intraspecific morphological variation among specimens from different host species, a potential geographical differentiation of *C. casuarinus* was tested solely on specimens collected from *H. stenosoma*. The acquired morphological data were combined with the previously published measurements on *C. casuarinus* ex *H. stenosoma* from the northern basin of Lake Tanganyika (see Table S1 and [31], raw data available in Mendeley Data doi:10.17632/ntjy37jwf3.1). The total dataset consisted of parasite specimens from three different localities in the northern basin (off Bujumbura, Uvira, and near the Malagarasi river delta) and one in the southern basin (off Mpulungu) (Figure 1). Intraspecific morphological variation was explored using principal component analysis (PCA) performed on scaled measurements from the haptor in the R package ade4 [48]. Additionally, host size, available for the specimens collected in Mpulungu, was visualised in biplots to test for a relationship between the patterns of morphological variation and host size. Missing data points were replaced by the average value and specimens with more than 50% missing measurements were excluded from the analysis. To test the significance of intraspecific differences in MCO structures, pairwise *t*-tests were performed in the R package stats [49]. The assumptions of normality and homogeneous variance within sample groups were verified by Levene’s test in R package stats [49].

#### 2.2.2. Geomorphometrics

Phenotypic variation of *C. casuarinus* related to geographic origin was also studied by shape analysis. The shape of the dorsal and ventral anchor of each parasite individual was digitised using eight fixed landmarks and 92 equally distributed semi landmarks (see Figure S1) in tps Dig v2.30 [50]. To minimise bending energy with respect to a mean reference form, fixed landmarks were superimposed using Generalized Full Procrustes Analyses under the Least Squares criterion [51,52]. PCA using fixed landmarks only was performed in MorphoJ v2.0 [53]. An ANOVA with a permutation test of 10,000 iterations was used to statistically validate differences between populations and dependency on the centroid size. A Relative Warp Analysis (RWA) [54] was performed on the overall shape of both anchors (using fixed landmarks and semi-landmarks) with the Procrustes coordinates using tps Relw v1.49 [50]. In order to give all landmarks equal weight, the scaling option was set to α = 0. Results of all multivariate statistics were visualised using the R packages ggplot2 [55] and tidyverse [56].

### 2.3. Genetics

#### 2.3.1. Data Acquisition

Whole genomic DNA extraction of the individual parasites (*n* = 26) (see Table 1) was performed using an optimised protocol for low input DNA samples. Initial digestion was performed in 195 µL of TNES buffer (400 mM NaCl, 20 mM EDTA, 50 mM Tris pH 8, 0.5% SDS) and 5 µL of proteinase *K* (20 mg/mL) incubated at 55 °C for ~30–60 min. DNA was precipitated in a mixture of 65 µL 5 M NaCl and 290 µL 96% EtOH yeast tRNA as a carrier, stored in −20 °C for 1 h and purified with three runs of 1 mL chilled 70% EtOH. Extracted DNA was eluted in 50 µL of 0.1× TE buffer with 0.02% Tween-20. To assess the intraspecific genetic diversity of *C. casuarinus* across Lake Tanganyika, part of the mitochondrial *cox1* gene was amplified using the primers ASmit1 (5′-TTT TTT GGG CAT CCT GAG GTT TAT-3′) [57] combined with Schisto3 (5′-TAAT GCAT MGG AAA AAA ACA-3′) [58], and with ASmit2 (5′-TAA AGA AAG AAC ATA ATG AAA ATG-3′) in a nested PCR [57]. The reaction mix for the first PCR contained one unit of Q5 High Fidelity Polymerase (New England Biolabs, Ipswich, MA, USA), 5× buffer containing 2 mM MgCl_2_, 0.2 mM dNTPs, 0.5 mM of the primers ASmit1 and Schisto3, 0.1 mM of bovine serum albumin (BSA) and 1 μL of isolated DNA (concentration was not measured) in a total reaction volume of 25 µL. The PCR was carried out under the following conditions: initial denaturation at 95 °C for 5 min, then 40 cycles of 1 min at 94 °C, 1 min at 55 °C and 1 min at 72 °C, and final elongation for 7 min at 72 °C. The nested PCR with ASmit1 and ASmit2 primers followed the same protocol as the first one with a 1:100 dilution of template DNA. To genetically verify parasite species identification for the new host-parasite combination reported in this study, individuals of *C. casuarinus* collected from *B. graueri* were further subjected to PCR of the 28S rRNA gene (28S), a nuclear marker traditionally used to help delineate monogenean species. Partial 28S was amplified using the primers C1 (5′-ACC CGC TGA ATT TAA GCA T-3′) and D2 (5′-TGG TCC GTG TTT CAA GAC-3′) [59]. Each reaction mix contained one unit of Q5 High Fidelity Polymerase (New England Biolabs, Ipswich, MA, USA), 5× buffer containing 2 mM MgCl_2_, 0.2 mM dNTPs, 0.5 mM of each primer and 2 μL of isolated DNA (concentration was not measured) in a total reaction volume of 30 μL. The PCR was conducted under the following conditions: 2 min at 94 °C, 39 cycles of 20 s at 94 °C, 30 s at 63 °C and 1 min and 30 s at 72 °C, and finally 10 min at 72 °C. The final PCR products were enzymatically purified using 4 µL of ExoSAP-IT reagent (ThermoFisher Scientific, Waltham, USA) and 10 µL of PCR product under the following conditions: 15 min at 37 °C and 15 min at 80 °C. Sequencing on an ABI3130 capillary sequencer was outsourced (Macrogen Europe). Electropherograms were visually inspected and corrected, and sequences were aligned using the Clustal Omega algorithm under eight threads [60] in Geneious Prime 2021.1.1 (https://www.geneious.com, accessed on 1 January 2021). The newly obtained haplotype sequences were deposited in NCBI GenBank under the accession numbers MZ384380-1 (28S rRNA) and MZ379290-MZ379315 (COI mtDNA).

#### 2.3.2. Population Genetic Analyses Based on *cox*1 Data

The obtained *cox*1 sequences (*n* = 26) were combined with the previously published sequence data of *C. casuarinus* from the northern basin (*n* = 42). The length of this combined alignment was 392 bp. The number of haplotypes and polymorphic sites, haplotype diversity, nucleotide diversity and Tajima’s D [61] were calculated in Arlequin v3.5 [62]. Phylogenetic relationships among haplotypes were inferred by constructing a Median Joining haplotype network in PopART v1.7 [63]. Population differentiation between parasite populations originating from the northern and southern basins was estimated by F_ST_ [64] as implemented in Arlequin v3.5 [62].

### 2.4. Genomics

#### 2.4.1. Mitogenome Assembly and Annotation

In total, 80 individuals of *C. casuarinus* ex *H. stenosoma* were pooled prior to DNA extraction to ensure a sufficient amount of high-quality DNA. Genomic DNA was extracted using the Quick-DNA^TM^ Miniprep Plus Kit (Zymo Research) following the manufacturer’s instructions with minor modifications, initial incubation overnight, and elution in 2 × 50 µL after 10 min incubation at room temperature each. Library preparation (Illumina TruSeq Nano, 550 bp insert size) and sequencing on the NovaSeq6000 (2× 150 bp) platform were outsourced (Macrogen Europe). Raw sequences were trimmed using Trimmomatic v0.39 [65] using a sliding windows option, cutting 5 bases from the start of each read and applying a minimal read length of 100 bp [65]. The mitogenome of *C. casuarinus* was assembled using part of the *cox*1 sequence (KX007864.1) as seed in NOVOPlasty v3.7.2 [66] with a k-mer length from 21–39, read length of 130 and insert size of 390. Partly assembled mitogenome sequences from k-mers 35 and 37 were combined in overlapping regions in Geneious Prime 2021.1.1 (https://www.geneious.com, accessed on 1 January 2021). The mitogenome was annotated using the MITOS web server (code echinoderm mitochondrial) [67] combined with visualisation of open reading frames and alignment with available mitogenomes of closely related monopisthocotylean monogeneans in Geneious Prime 2021.1.1 (https://www.geneious.com, accessed on 1 January 2021). In addition to MITOS, the tRNAscan-SE [68] and RNAfold [69] web servers were used to verify the tRNA-coding regions. When results between applications conflicted, the solution proposing a 7 bp acceptor stem was chosen. As non-coding mitochondrial regions were assembled, the presence of repeat sequences was checked with Tandem Repeats Finder [70]. Raw Illumina reads were submitted to SRA (accession: SRR15217800) under BioProject accession PRJNA749051.

#### 2.4.2. Mitogenome Diversity

Trimmed reads were mapped back to the assembled mitogenome, both majority consensus sequences, respectively, using bwa mem with the mean insert size of 450 bp (min. 300 bp, max. 1000 bp) [71]. Bwa mem has been identified as a suitable alignment method due to low false-positive rates and has been demonstrated to be the most effective open-source method for mapping PoolSeq data [72]. PCR duplicates were removed using SAMBLASTER v0.1.24 [73]. Mapped reads were filtered for low quality (-q 20) and paired reads only with SAMtools v0.1.11 [74]. The resulting bam files were converted to mpileup files using SAMtools v0.1.11 [74]. The number of polymorphic sites, nucleotide diversity (π) and Tajima’s D in the pooled sample were calculated in PoPoolation v.1.2.2 [75] using a sliding window approach across the mitogenome excluding the repeat region (window size of 300 bp and a step size of 10 bp, minimum coverage 4, minimum count 2) and across the *cox*1 fragment (window size of 392 bp and a step size of 2 bp, minimum coverage 4, minimum count 2, pool size 80 and minimum coverage fraction 0.6). To assess the interspecific nucleotide diversity between the mitochondrial protein-coding genes known from species in this parasite genus, a sliding window analysis was performed on aligned sequences of two other species of *Cichlidogyrus* (*C. halli* (Price & Kirk, 1967) MG970255.1 [76] and *C. sclerosus* Paperna & Thurston, 1969 JQ038226.1 (unpublished) and the majority rule consensus sequence of *C. casuarinus* in DnaSP v5 [77] (with a window size of 300 bp and a step size of 10 bp). These are the only two members of the genus for which a complete mitochondrial genome sequence was already available. Conveniently, *C. halli*, *C. sclerosus* and *C. casuarinus* belong to different clades within *Cichlidogyrus* [78], ensuring a certain phylogenetic coverage of the genus.

#### 2.4.3. Ribosomal Operon

To assemble the nuclear ribosomal operon, trimmed paired-end reads were baited (k = 31) using Mirabait v5 [79] based on the reference 28S (KX007821.1), 18S and ITS-1 rDNA (KX007795.1) sequences of *C. casuarinus*. The baited fraction of the reads were subjected to de novo assembly in SPAdes v3.15.1 [80]. K-mer lengths were set at 21, 33, 55, 77, 99 and 127. The Resulting de Bruijn graphs were visualised with Bandage v0.8.1 [81] and subjected to a BLAST search against the reference sequence. The respective positions of 18S, 28S and 5.8S rRNA were predicted using RNAmmer v1.2 [82]. To identify the boundaries of the ITS1 and ITS2 regions, contigs were fed into ITSx v1.1.3 [83]. To confirm the gene boundaries, resulting contigs were aligned to the ribosomal operon of available species of *Cichlidogyrus* (*C. halli* MG973075.1 and *C. mbirizei* MG973076.1) using Muscle v3.8.435 under a max. number of 13 threads [84] in Geneious Prime 2021.1.1 (https://www.geneious.com, accessed on 1 January 2021). The annotated sequence of the ribosomal operon was deposited in NCBI GenBank under the accession number MZ700354.

## 3. Results

In total, 156 individuals of *C. casuarinus* were collected from *H. stenosoma* (prevalence 88.9%, maximum infection intensity 86, minimum infection intensity 1, mean 19.5, and abundance 17.3). Nine individuals of *C. casuarinus* were collected from *B. graueri*, representing the first record on this host. All the collected monogenean specimens were identified as *C. casuarinus* based on the morphological details of haptoral structures (mainly length of dorsal bar and first pair of marginal hooks), the fact that the male copulatory tube has a spirally thickened wall, and that the MCO has a 26–59-µm-long heel.

### 3.1. Morphological Variation

Based on the observed mutual position of parasitic individuals in the PCA scatterplot, phenotypic variation related to geographic origin was visible along the first and second PC axes (Figure 1a). The pattern was driven mainly by the ‘total length’ and ‘length to notch’ of both anchors and the ‘maximum straight width’ of the dorsal bar (Figure 1a). Moreover, a clustering of specimens collected from similar-sized fish hosts was visible along the second PC axis. Conversely, no significant differences in the morphology of the parasite’s MCO related to basin were detected (copulatory tube length—F = 0.000_(1,65)_, *p* = 0.989, heel length—F = 0.132_(1,68)_, *p* = 0.718, Figure 1b,c).

**Figure 2:**
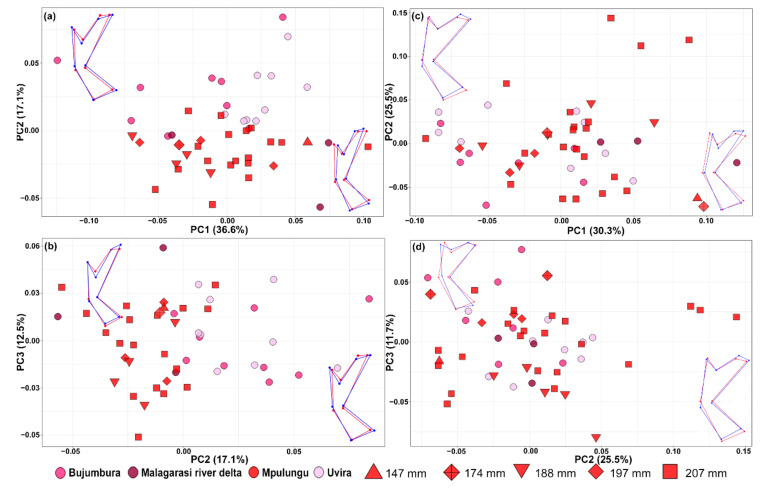
Biplots showing the shape variation in anchors of *Cichlidogyrus casuarinus* ex *Hemibates stenosoma*; shape changes along each PC are shown by wireframes with starting shapes (consensus, value 0) in red, and target shapes (changes) associated with extreme values (value +0.1) in dark blue. Only the first three axes are shown. (**a**,**b**) PCAs based on Procrustes distances of eight fixed landmarks describing the shape of the dorsal anchor. (**c**,**d**) PCAs based on Procrustes distances of eight fixed landmarks describing the shape of the ventral anchor.

**Figure 3:**
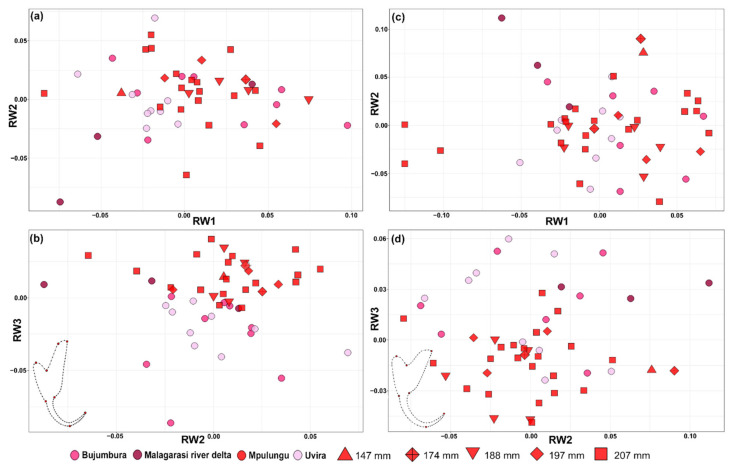
Biplots showing the shape variation in anchors of *Cichlidogyrus casuarinus* ex *Hemibates stenosoma*. Only the first three axes are shown. (**a**,**b**) RWA of the dorsal anchor using a semi-landmark sliding approach. (**c**,**d**) RWA of the ventral anchor using a semi-landmark sliding approach. Consensus shape displaying the position of fixed (red dots) and semi-landmarks (black dots) in dorsal and ventral anchors, respectively, is shown.

Overall, a more pronounced differentiation between geographically defined populations was apparent in the shape of the dorsal anchor compared to the ventral one (Figure 2). Differentiation of geographically defined populations of *C. casuarinus* in the shape of the dorsal anchor was visible mainly along the second PC axis. The results of RWA (including sliding landmarks) followed the pattern obtained via PCA but did not provide a higher resolution (Figure 3). Nevertheless, the shape of both anchors is significantly different between the basins (dorsal anchor—F = 4.8_(12,552)_, *p* < 0.0001, ventral anchor—F = 2.39_(12,588),_ *p* = 0.0051) with significant correlation between the shape and centroid size of dorsal (F = 52.52_(1,46)_, *p* < 0.0001) and ventral anchor (F = 28.93_(1,49)_, *p* < 0.0001), respectively.

### 3.2. Genetic Diversity and Differentiation

**Figure 4:**
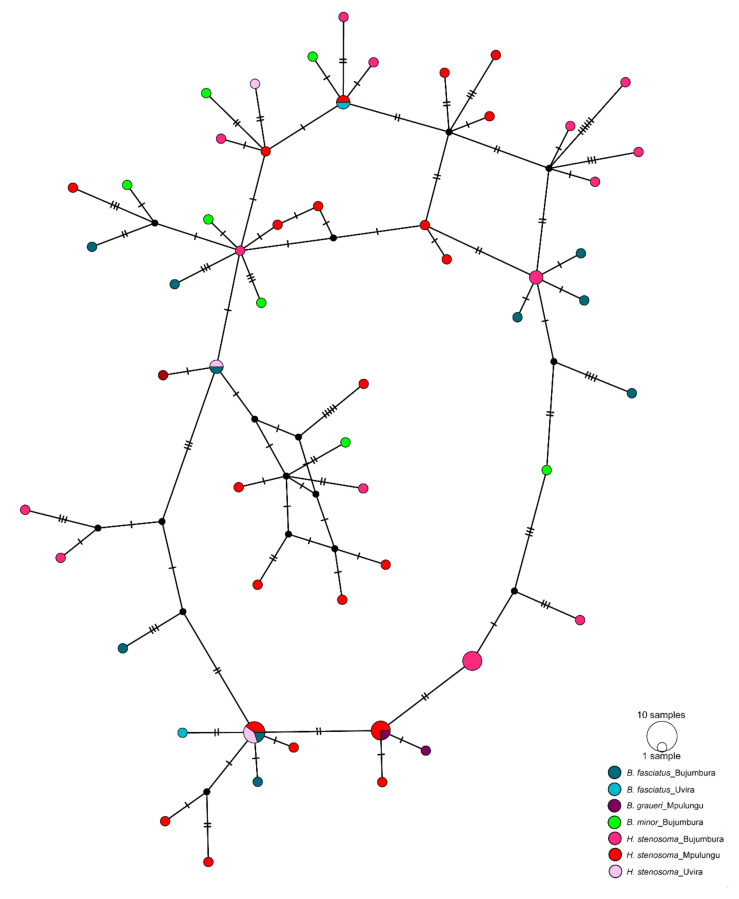
Genetic population structure of *Cichlidogyrus casuarinus* from Lake Tanganyika, East Africa, based on the mitochondrial cytochrome *c* oxidase subunit I (*cox*1) sequences. Median-joining haplotype network for worms recovered from four species of Bathybatini (see legend) at several locations across Lake Tanganyika. Coloured circles represent observed haplotypes where the size is proportional to the number of individuals sharing a haplotype.

New sequences for *cox*1 mtDNA were obtained from 24 individuals of *C. casuarinus* ex *H. stenosoma* from the southern basin (Mpulungu), comprising 21 different haplotypes and containing 33 polymorphic sites. Genetic distances among haplotypes ranged from 0.3% to 3.8%. Haplotype and nucleotide diversity in the southern basin was estimated at 0.987 and 0.02017, respectively. Tajima’s D was negative, but not significantly different from zero (D = −0.39985, *p* = 0.39800). Overall, the total dataset (lake-wide sample, including previously published data) of the *cox*1 gene portion (*n* = 68) comprised 55 different haplotypes with 65 polymorphic sites. Haplotype and nucleotide diversity was estimated at 0.9890 and 0.021099, respectively. Tajima’s D was negative, but not significantly different from zero (D = −1.31540, *p* = 0.07300). The non-hierarchical topology of the haplotype network indicated the absence of a geographically driven population structure (Figure 4). However, significant differentiation between populations from *H. stenosoma* from the northern basin and Mpulungu (the southern basin) (F_ST_ = 0.05002, *p* = 0.04524 ± 0.0020) was observed.

### 3.3. Mitogenome and Nuclear Ribosomal Operon

**Figure 5:**
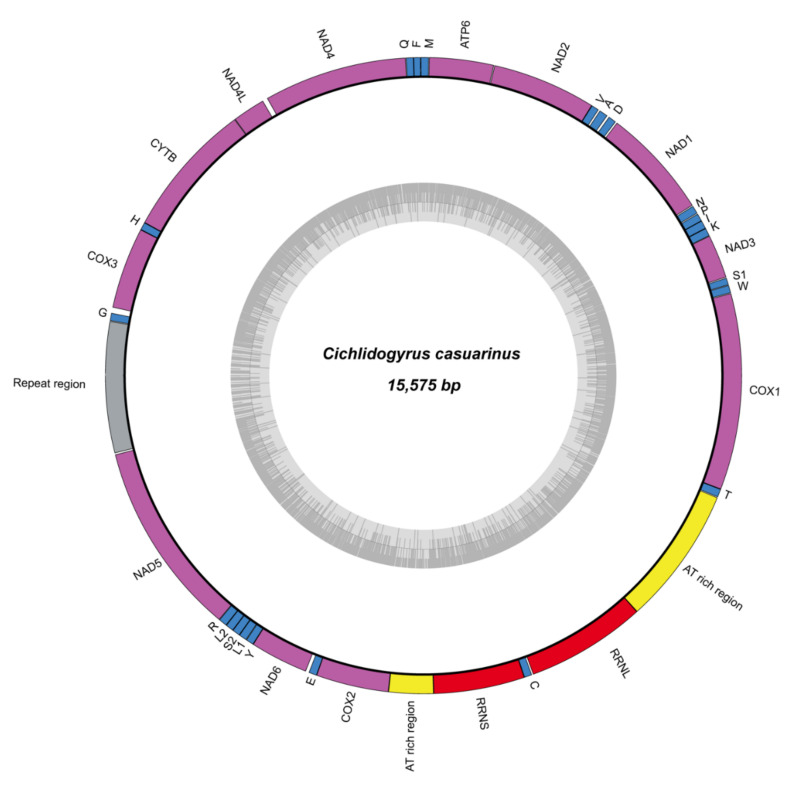
The mitochondrial genome of *Cichlidogyrus casuarinus* ex *Hemibates stenosoma* with GC content (inner circle).

Genomic DNA sequencing on the Illumina NovaSeq6000 platform yielded 15,980,972 indexed paired-end reads. A complete mitochondrial genome of 15,575 bp was assembled and annotated (Figure 5). The total number of properly mapped reads across the assembled mitochondrial genome was 76,009 after filtering steps. The coverage along the various mitochondrial regions is detailed in Table 2. The mitochondrial genome of *C. casuarinus* comprises 12 (all except *atp*8) intron free protein coding genes, 22 tRNA genes and two genes coding for the large and small subunits of the mitochondrial rRNA following the gene order of other species of *Cichlidogyrus* (see Table 2) [76]. The absence of *atp*8 was detected as in other neodermatan flatworms [85]. We report an abbreviated stop codon TA for *nad*2 as previously observed in *C. halli* and *C. mbirizei* [76]. An alternative start codon ATT was found for *nad*1. Three non-coding regions (NCRs) were assembled in the mitochondrial genome of *C. casuarinus*. One of the non-coding regions is located before the genes coding for rRNA and is AT-rich (1096 bp, 31.8% GC). Further, another AT-rich region was assembled after the genes coding for rRNA (354 bp, 21.1% GC). A repeat region of 1307 bp long including 11 repetitions of a 90 bp motif is located between the genes coding for *nad*5 and *trn*G.

**Figure 6:**
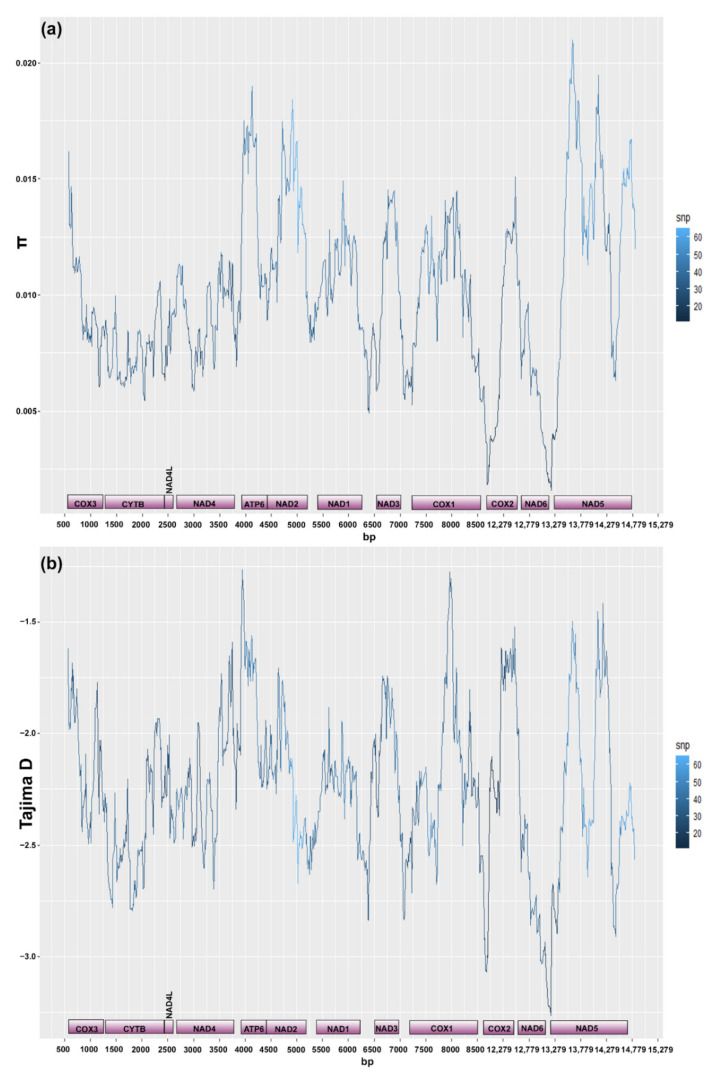
Sliding window analyses (window size 300 bp, step size 10 bp) of PoolSeq data to infer nucleotide diversity (**a**) and Tajima’s D parameter (**b**) across the mitogenome of *Cichlidogyrus casuarinus* ex *Hemibates stenosoma* (excluding ribosomal and non-coding genes). Gene boundaries with the respective position in the mitogenome are below the graph. The colour scale denotes the number of SNPs in the sliding window.

**Figure 7:**
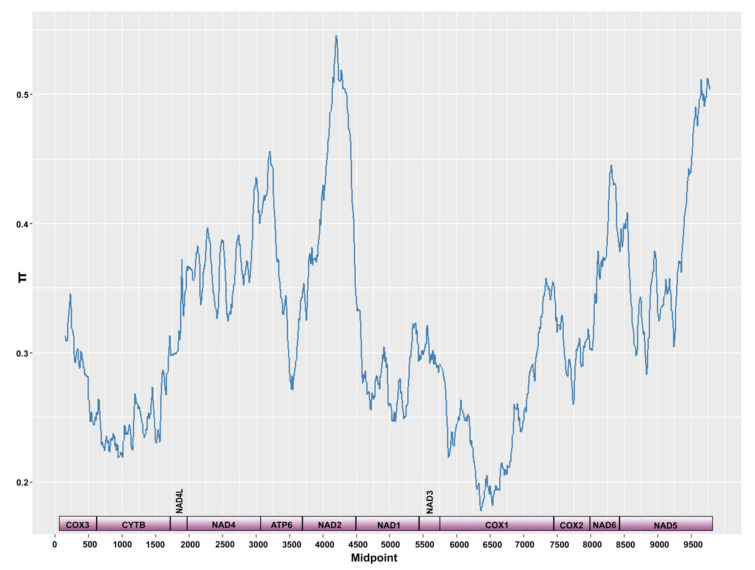
Sliding window analysis (window size 300 bp, step size 10 bp) of the alignment of mitochondrial protein-coding genes of the three complete mitochondrial genomes of *Cichlidogyrus* spp. (*C. casuarinus* MZ703276, *C. halli* MG970255.1 and *C. sclerosus* JQ038226.1). The line indicates the nucleotide diversity with gene boundaries indicated below the graph.

The sliding window analysis showed varying levels of intraspecific nucleotide diversity between the protein coding genes of *C. casuarinus* with the highest values reported for *atp*6, *nad*2 and parts of *nad*5 (see Figure 6a). All protein-coding genes showed negative values of Tajima’s D with the lowest values in *cyt*b and *nad*6 (Figure 6b). The nucleotide diversity for the *cox*1 fragment in the PoolSeq data was 0.01460, Tajima’s D parameter −1.67146. In contrast to the intraspecific level, at the interspecific level, the gene coding for *cox*1 showed the lowest level of nucleotide diversity in comparison to other protein coding genes. The highest values were reported for the *nad*2 and *nad*5 genes (Figure 7).

**Figure 8:**
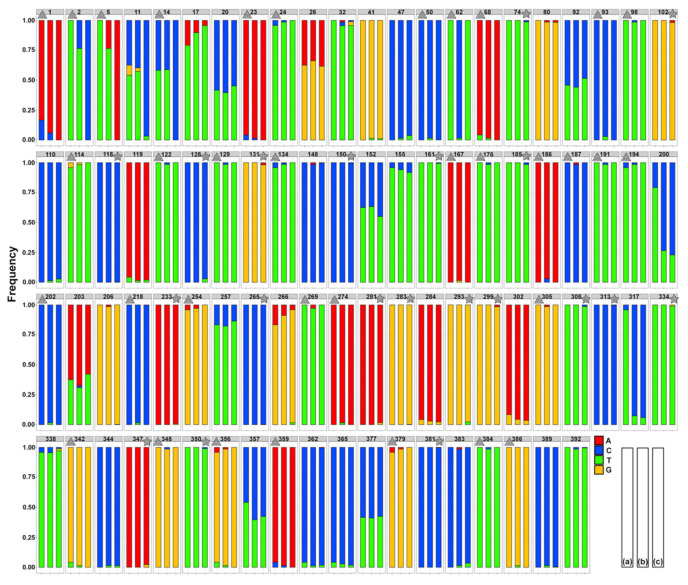
Distribution of allele frequencies across all captured polymorphic sites in the *cox*1 gene portion of *Cichlidogyrus casuarinus* in (**a**) Individual-based sequences ex *Hemibates stenosoma*, Mpulungu, September 2018, (**b**) Individual-based sequencing including all sequence data of *C. casuarinus* available, (**c**) Pooled NGS dataset ex *H. stenosoma*, Mpulungu, September 2018. Polymorphic sites unique to individual-based or NGS datasets are marked with triangle and asterisk, respectively.

In comparison to 33 (*C. casuarinus* ex *H. stenosoma*, Mpulungu) and 65 (all *C. casuarinus* samples), polymorphic sites reported in the Sanger sequencing-based datasets, 51 SNPs were identified in the PoolSeq data across the respective portion of *cox*1 gene (392 base pairs). The number of unique polymorphic sites was 13 in the individual-based from Mpulungu only, and 20 in the PoolSeq data, respectively. The number of shared polymorphic sites (SNPs) between individual-based and pooled datasets collected in Mpulungu, September 2018 was 18, i.e., 35% of PoolSeq and 55% of Sanger-based SNPs. The lowest allele frequency captured by the individual-based dataset was 0.0147 compared to 0.0024 in the NGS dataset. A comparison of the allele frequency distributions across all polymorphic sites and the strong agreement between methods is shown in Figure 8.

The total length of the ribosomal operon was 7014 bp (see Table S2). The uncorrected p-distance between assembled rDNA regions of *C. casuarinus* and *C. halli* including gaps was 1.32% in 18S rDNA, 29.09% in ITS1, 0.64% in 5.8 rDNA, 60.54% in ITS2 and 2.65% in 28S rDNA. The uncorrected p-distance between assembled rDNA regions of *C. casuarinus* and *C. mbirizei* including gaps was 0.74% in 18S rDNA, 29.78% in ITS1, 0% in 5.8 rDNA, 37.78% in ITS2 and 2.27% in 28S rDNA.

## 4. Discussion

### 4.1. Morphological and Mitochondrial Diversity and Host Use in Cichlidogyrus casuarinus

In this study, phenotypic variation related to the geographic origin of a monogenean parasite infecting bentho- and eupelagic fish hosts in Lake Tanganyika, *C. casuarinus*, was contrasted with the lack of a clear phylogeographic structure in the genetic data. In a deepwater pelagic environment under the absence of apparent physical barriers and offering only a limited number of ecological niches, fish speciation is assumed to be mainly driven by resource partitioning [86,87] and spawning behaviour [22,88,89,90]. Therefore, benthopelagic foraging is suggested to limit the dispersal propensities of *B. graueri* and *H. stenosoma*, which stands in stark contrast to the unrestricted migration of eupelagic species such as *Bathybates fasciatus* Boulenger, 1901 and *Bathybates leo* Poll, 1956. The lack of clear phylogeographic structure in *C. casuarinus* contrasts with the reported north-south gradient seen in the host species, *H. stenosoma* [45]. We propose that this is a result of the parasite’s intermediate generalist lifestyle [38] infecting a range of host species from different genera. Purely pelagic host species of *C. casuarinus*, such as *B. fasciatus* and *B. leo* [31,39], show no restriction of gene flow in the study of [45]. These species seem to migrate on a lake-wide scale, and may hence transport *C. casuarinus* across Lake Tanganyika. Therefore, our results show a limitation of the parasite’s magnifying potential caused by the least structured and the most mobile host species, as these are even in low densities sufficient to maintain a widely distributed and lake-wide nearly panmictic parasite population. This suggests that, despite strong interspecific niche segregation between hosts regarding prey preferences, main habitat use, and preferred water depth [91], bathybatine host species (at least seasonally) overlap in their occurrence under conditions favourable for monogeneans to switch back and forth between the different host taxa. Whether bathybatines also occur in mixed shoals like semi-pelagic cichlids of the more shore associated genera *Cyprichromis* and *Paracyprichromis* [29,92] is not known for sure, but mixed catches at the local fish markets indicate that they do occur, at least occasionally, together. Alternatively, as the monogenean-host relationships in Lake Tanganyika remain poorly understood [78], undescribed host interactions of *C. casuarinus* could bridge physical niche partitioning of bathybatine cichlids. High levels of haplotype and nucleotide diversity in the studied portion of the *cox*1 region concur with previously reported results for *C. casuarinus* in the northern part of the lake. Further, negative values of Tajima’s D, though not significant, are consistent with previously suggested population expansion in this monogenean species congruent to the demographic history of some of the bathybatine hosts [45].

In general, as a crucial part of the attachment organ and the physical interface between parasite and host, sclerotised haptoral structures of monogeneans are presumably under strong evolutionary constraints [93,94]. Given the previously reported lack of host preference within the northern basin [31] and the absence of a clear geographic structure on a lake-wide scale found in the present study, we propose that geographical morphological variation displayed by *C. casuarinus* is driven by external environmental conditions imprinted during ontogeny. Specifically, the variation present in the dorsal anchor and bar of *C. casuarinus* correlates with both host species identity [31] and environmental conditions related to different geographic origins (present study). The present study reveals that morphological variation in the ventral anchor is related to geography in *C. casuarinus* ex *H. stenosoma*. Moreover, geographically dependent morphological variation in both anchors was reported for *Cichlidogyrus milangelnari* Rahmouni, Vanhove & Šimková, 2017 infecting the Lake Tanganyika cichlid *Cyprichromis microlepidotus* (Poll, 1956) [37]. In general, the overall shape of the ventral anchor was found to be more informative for the host species identity [31] and the dorsal anchor for the external environment. The lack of a clear genetic phylogeographic structure in *C. casuarinus* is in accordance with the fact that the MCO structures are of similar size throughout the lake, suggesting there is no reproductive isolation at this geographical scale.

A correlation between host dispersal capacity and monogenean intraspecific morphological variability was suggested already for other cichlid-monogenean combinations in the lake by another recent study [37]. Similar to the present study, morphological variation related to different hosts and external environmental conditions was reported in *Neobenedenia girellae* (Hargis, 1955), a cosmopolitan fish parasite [95] and *Gyrodactylus katharineri* Malmberg, 1964 infecting various cyprinid hosts in Europe [96]. In combination with our present findings, this suggests that elevated levels of phenotypic plasticity comprising a wide range of morphological characters are expected in rather generalist monogenean species, including *C. casuarinus,* compared to more specialised congeners such as *Cichlidogyrus irenae* Gillardin, Vanhove, Pariselle, Huyse & Volckaert, 2012 infecting *Gnathochromis pfefferi* (Boulenger, 1898) (e.g., the total length of the dorsal anchor 40–73.8 μm versus 27–37.5 μm, inner root length of dorsal anchor 15.5–31.3 μm versus 19.8–23.9 μm, the total length of ventral anchor 38.3–62.5 μm versus 26.9–36.4 μm and inner root length of ventral anchor 10.1–21.6 μm versus 5.6–10.8 μm) [97]. This pattern has also been reported for other monogenean genera such as *Kapentagyrus* infecting clupeid species in Lake Tanganyika [98] or *Lamellodiscus* spp. from sparid fishes in the Mediterranean Sea [99]. Moreover, the previous suggestion that host species size is related to morphological variation of *C. casuarinus* [31] has been confirmed here, and similar results were already reported for *Kapentagyrus* spp. infecting clupeid species in Lake Tanganyika [36] and *Kuhnia scombri* (Kuhn, 1829) parasitizing *Scomber australasicus* Cuvier, 1832 and *S. japonicus* Houttuyn, 1782 in the Indo-Pacific Ocean [100].

The known host range of *C. casuarinus* was updated with *B. graueri*. This host-parasite interaction was not found in the northern part of Lake Tanganyika [31], which may be explained by geographically and/or seasonally dependent infection patterns of *C. casuarinus* on *B. graueri*. However, given the overall low level of geographic structuring and the lack of host preference, it is also possible that this interaction was missed due to a rather low number of available host specimens in this previous study. Consequently, only two species of Bathybatini remain unconfirmed for the presence of monogenean parasites, *Bathybates ferox* Boulenger, 1898 (investigated in [31], but only *n* = 7) and *Hemibates koningsi* Schedel & Schliewen, 2017, the latter of which was only described recently [101].

### 4.2. Ribosomal Operon and Its Utility for Future Studies/Research

Portions of the ribosomal operon coding for the nuclear ribosomal RNA (18S, 5.8S and 28S rRNA) and internal transcribed spacer regions (ETS, ITS1, ITS2) are widely used for inferring phylogenetic relationships among parasitic [58,102] as well as free-living flatworms [103]. However, the low number of species with an available assembled ribosomal operon has restricted its full use in inferring phylogenetic relationships among monogenean taxa so far. Within parasitic flatworms, the combination of rRNA genes and ITS regions is commonly applied to address species level boundaries [102,104]. Length differences of the ribosomal operon among species of *Cichlidogyrus* (7,496 bp in *C. halli*, 7,005 bp in *C. mbirizei* and 7,014 bp in *C. casuarinus*) are mainly present in the ITS regions, as reported in the first genomic study on African monogeneans [76]. Gaining knowledge about the interspecific variation present at this multi-copied DNA locus could be further applied to the emerging field of environmental (eDNA) metabarcoding and metagenomics and enable the routine identification of parasite communities including monogeneans.

### 4.3. Mitochondrial Genome

#### 4.3.1. Lake Tanganyika and the Rest of the Monogenean World

In the present study, the first monogenean and first parasite mitochondrial genome from Lake Tanganyika is presented. A high level of genomic diversity in mitochondria including numerous rearrangements has been previously reported in monogeneans [76], other parasitic or endosymbiotic [105] and free-living flatworms [106]. Comparisons at the family level of Dactylogyridae revealed tRNA gene transposition of *trn*T and between *trn*L2 and *trn*R (reviewed in [85]). Unlike in other genera of parasitic flatworms, such as *Schistosoma* spp. [107] or *Syndesmis* spp. [105], the lack of rearrangements of the order of protein coding genes (PCGs) or tRNA genes compared to other species of *Cichlidogyrus* suggests that gene order is conserved in this monogenean lineage across different clades [78]. Similar to its congeners for which the full mitogenome is available, three non-coding regions were assembled in *C. casuarinus*. Variability in the position, length and GC content in NCRs within and between lineages was previously reported in endosymbiotic/parasitic [76,105] and also free-living flatworms [108]. However, the presence of an NCR between the *nad*5 and *cox*3 coding genes has been found in all representatives of Dactylogyridae and in other monogenean families such as Diplectanidae [109] and Tetraonchidae [110] for which the mitochondrial genomes are available. Similarly, the position of an AT-rich NCR between *rrn*S and *cox*2 seems to be fixed in *Cichlidogyrus,* as already suggested by [76]. However, as more than 130 species of *Cichlidogyrus* have been already described [27], future mitogenomic studies are needed to verify the generality of these patterns. In the mitogenome of *C. casuarinus*, the position of an AT-rich NCR between *rrn*L and *cox*1 is currently unique within monogeneans. Mitochondria play a central role in energy generation and in several other mechanisms involved in cellular homeostasis [111]. The function of NCRs in the mitogenomes of flatworms remains for the most part unknown but a function in mtDNA replication and transcription, including the initiation site for replication, has been suggested [112,113]. Most of the assembled PCGs in the mitogenome of *C. casuarinus* employed canonical start and stop codons, but similarly to the situation in other species of *Cichlidogyrus*, *cox*3 and *nad*2 regions end in abbreviated stop codons T and TA, respectively. However, in the case of *nad*2, an overlap of 1 bp with *trn*V would allow the presence of the canonical stop codon TAG as was reported in the annotation of *C. sclerosus* (JQ038226, unpublished). Truncated stop codons have been reported across different lineages of parasitic [76,85,114], endosymbiotic [105] and free-living flatworm taxa [115], and also in early diverging acoelomorphs [116]. An alternative start codon ATT was previously assembled for several flatworm taxa [105,108,117,118] but here it is reported for the first time in dactylogyrid monogeneans, as the start codon of the *nad*1 gene in *C. casuarinus*.

#### 4.3.2. Intraspecific Variation at the Mitogenome Level

In concordance with previous studies on monogenean mitochondrial diversity [76,119], the *cox*1 region appeared as the least variable PCG at the interspecific level. Moreover, it showed the lowest non-synonymous to synonymous substitution ratio compared to the other PCGs in previous studies [76,110] and its product is considered a highly conserved protein [120]. Moreover, the reported negative values of Tajima’s D across the mitogenome of *C. casuarinus* suggest that all PCGs are under purifying selection and/or the species experienced recent population expansion [61]. The sliding window approach applied on pooled NGS data of *C. casuarinus* (Figure 6) revealed a similar level of nucleotide diversity in *cox*1 as in the other PCGs. Globally, purifying selection which acts against deleterious mutations is reported for mitogenomes across the animal kingdom in line with the major role of mitochondria in the respiratory chain which requires coding sequence functionality [121]. However, purifying selection acting on mitochondrial genes does not prevent local positive selection at the intraspecific level driven by host and/or environmental differences with the highest number of polymorphic sites occurring in *cox*1 and *cyt*B [122]. In comparison to other pelagic monogenean lineages in Lake Tanganyika, such as *Kapentagyrus* spp. infecting clupeids and *Dolicirroplectanum lacustre* (Thurston and Paperna, 1969) parasitising on lates perches, *C. casuarinus* showed a higher nucleotide diversity in *cox*1 [36,123]. Adaptive evolution driven by life-history innovations acting on mitochondrial genes has been already reported for monogeneans [124], other parasitic flatworms and other invertebrate and vertebrate taxa [125], including cichlid fishes in Lake Tanganyika [126]. The high level of intraspecific variation in the *cox*1 region might be explained by the generalist lifestyle of *C. casuarinus*, possibly as an adaptation to the broad ecological niche of its host assemblage.

High coverage in regions coding for rRNA (Table 2) might be explained by the uneven *post-mortem* fragmentation of mitochondrial regions resulting in uneven representation in genome libraries towards better-preserved regions [127] or by certain motifs being prone to high rates of error and low coverage [128]. Alternatively, the presence of nuclear insertions of mitochondrial origin (NUMTs) as detected in flatworms [129] and nuclear genomes of various organisms [130,131] cannot be excluded.

#### 4.3.3. Methodological Implications for Future Studies

Notably, allele frequencies in the shared polymorphic sites identified using the individual-based approach and PoolSeq dataset, respectively, were highly comparable. We report a higher number of polymorphic sites (51 vs. 33) and lower nucleotide diversity in the pooled NGS data compared to the individually retrieved haplotypes of the *cox*1 gene portion retrieved from the same metapopulation of *C. casuarinus*. These results correspond with the larger number of individuals pooled compared to individually sequenced (80 vs. 24) and the relatively high haplotype diversity in the studied *cox*1 gene portion of *C. casuarinus*. As such, the reported minor differences in allele frequencies between individual-based and NGS datasets might be a consequence of the different parasite individuals the data were generated from. False-positive SNPs can be possibly identified using the known frequency of the rare alleles present in the population of targeted species as a threshold [132]. In our study, 10 polymorphic sites unique to the NGS dataset showed a lower frequency compared to the rarest allele captured using individual-based sequencing (see Figure 8) and could be therefore considered as false positive. The unique sites showed the relatively low frequency of the alternative allele. Additionally, nine SNPs in the PoolSeq data had a lower frequency compared to the theoretical threshold of a singleton in a population of 80 individuals (allele frequency of 0.0125). The absence of certain polymorphic sites (13 captured in the individual-based approach from Mpulungu only) could be caused by the loss of rare haplotypes due to the necessary filtering steps as part of the NGS data pipeline [133,134]. The reported difference might be further related to the lower coverage per individual (ranging from 7.6 to 13.6× see Table 2) compared to the 20× proposed to adequately reflect genetic variability based on experimental studies [135,136]. Additionally, DNA quantification followed by optimisation of DNA input per specimen prior to pooling might reduce the bias towards certain specimens and sites [134,136].

## 5. Conclusions

Population-genetic patterns of parasites often represent an enhanced reflection of their hosts’ population structure due to their fast generation time, but this rule of thumb may be hampered by the unrestricted gene flow among parasite populations with weak host specificity. Here, we tested this caveat using the intermediate generalist monogenean flatworm *C. casuarinus* from populations across whole Lake Tanganyika. We found that although morphological differences occurred between specimens of different localities, the apparent genetic structure between populations was missing. Our findings are consistent with the previously reported host driven morphological variation without genetic differentiation of these monogeneans, and highlight the importance of integratively approaching the parasites’ potential as “tags” for their hosts. Moreover, our results show a limitation of the parasite’s magnifying potential by the least structured and the most mobile host species, as these are even in low densities sufficient to maintain a widely distributed and lake-wide almost panmictic parasite population. The maintenance of the generalist lifestyle of *C. casuarinus* might be explained by its adaptation to low host availability via stabilizing selection on genotypes promoting relatively high morphological variation. However, more data are needed to reveal the processes behind the recorded patterns. Population genomics on monogeneans, sourced from the wild without experimental procedures, is to our knowledge an uncovered field. Our study presents the first parasite mitogenome from Lake Tanganyika and suggests a contrast between intra- and interspecific variation in mitochondrial PCGs within *Cichlidogyrus*. Overall, PoolSeq proved to be a suitable approach to assemble the mitogenome of tiny non-model organisms preserved under field conditions, and to evaluate the level of intraspecific nucleotide diversity across the mitogenome.

Given its relatively high abundance, and the now considerable baseline knowledge on its morphological and genetic variation, combined with a widespread occurrence in the closed pelagic ecosystem of Lake Tanganyika, we propose *C. casuarinus* as a model to study (1) mechanisms driving host-range difference in comparison with host-specific species of *Cichlidogyrus* that also occur in Lake Tanganyika, and (2) the role of phenotypic plasticity in (the lack of) diversification and speciation.

## Figures and Tables

**Table 1:** Overview of specimens of *Cichlidogyrus casuarinus* being used in different parts of the study.

Host Species	Morphological Characterisation	Genetic Characterisation (*cox*1 mtDNA)	Genomic Characterisation (PoolSeq)
*Hemibates stenosoma*	43	24	80
*Bathybates graueri*	5	2	-

**Table 2:** Overview of the length (in bp) of mitochondrial regions, the start and stop codons (protein-coding genes), anticodons (tRNA genes), and minimum–maximum coverage in the assembled mitogenome of *Cichlidogyrus casuarinus* ex *Hemibates stenosoma* based on a pooled sample of 80 individuals.

Region	Position	Length	Start/Stop Codon	Anticodon	Min–Max Coverage
*trn*G	430–491	62		TTC	463–562
*cox*3	536–1181	646	ATG/T		571–803
*trn*H	1182–1243	62		GTG	690–763
*cyt*b	1244–2320	1077	ATG/TAG		620–818
*nad*4L	2322–2582	261	ATG/TAG		494–674
*nad*4	2621–3754	1134	GTG/TAG		411–750
*trn*Q	3757–3817	60		TTG	650–794
*trn*F	3816–3871	56		GAA	785–841
*trn*M	3872–3935	64		CAT	683–814
*atp*6	3938–4447	510	ATG/TAA		518–876
*nad*2	4452–5275	824	ATG/TA		609–916
*trn*V	5276–5337	62		TAC	780–833
*trn*A	5350–5419	70		TGC	771–831
*trn*D	5436–5500	65		GTC	758–799
*nad*1	5501–6388	876	ATT/TAA		415–860
*trn*N	6395–6460	66		GTT	677–815
*trn*P	6466–6528	63		TGG	800–867
*trn*I	6528–6594	67		GAT	820–878
*trn*K	6595–6659	65		CTT	767–822
*nad*3	6661–7008	348	GTG/TAG		688–774
*trn*S1	7014–7070	57		GTC	722–809
*trn*W	7073–7135	63		TCA	702–811
*cox*1	7139–8692	1554	ATG/TAA		602–1088
*trn*T	8693–8758	66		TGT	744–843
AT-rich region	8763–9858	1096			181–1701
*rrn*L	9859–10,809	951			1253–1800
*trn*C	10,820–10,881	62		GCA	1879–2213
*rrn*S	10,882–11,601	720			1069–2284
AT-rich region	11,602–11,955	354			590–1214
*cox*2	11,956–12,531	576	ATG/TAG		857–1298
*trn*E	12,531–12,595	65		TTC	821–965
*nad*6	12,621–13,082	462	GTG/TAG		606–800
*trn*Y	13,083–13,145	63		GTA	791–885
*trn*L1	13,147–13,211	65		TAG	879–921
*trn*S2	13,212–13,274	63		TGA	837–897
*trn*L2	13,275–13,339	65		TAA	792–881
*trn*R	13,340–13,407	68		TCG	657–815
*nad*5	13,409–14,953	1545	ATG/TAA		430–862
Repeat region	14,965–426	1037			12–829

## Data Availability

Parasite voucher material was deposited in the collection of Hasselt University under accession numbers XIV.1.16–1.50 and XIV.2.01–2.33. The morphometric and geometric morphometric data underlying the results of this article are available in Mendeley Data (doi:10.17632/ntjy37jwf3.1). The DNA sequence data are available in the GenBank Nucleotide Database under the accession numbers MZ384380-1 (28S rRNA), MZ379290-MZ379315 (COI mtDNA), MZ700354 (ribosomal operon) and MZ703276 (mitogenome). Raw Illumina reads were submitted to SRA (accession: SRR15217800) under BioProject accession PRJNA749051.

## Supplementary Materials

The following are available online at https://www.mdpi.com/article/10.3390/biology10080797/s1, Figure S1: Position of fixed landmarks (big points) as well as semi-landmarks (small points) on the dorsal anchor of *Cichlidogyrus casuarinus*. Number and position of landmarks was followed in the analyses of the ventral anchor, Table S1: Measurements of *Cichlidogyrus casuarinus* from Mpulungu. Zambia collected from *Hemibates stenosoma* and *Bathybates graueri*. respectively (a—mean value±standard deviation. b—range), Table S2: Overview of the length (in bp) of rDNA regions in the partly assembled ribosomal operon of *Cichlidogyrus casuarinus* based on a pooled sample of 80 individuals (GenBank accession number MZ700354), Table S3: Overview of the allele frequencies recovered from different datasets in the analysed fragment of *cox*1 gene (392 bp).
