# Supplementary material for: Contrasting Host-Parasite Population Structure: Morphology and Mitogenomics of a Parasitic Flatworm on Pelagic Deepwater Cichlid Fishes from Lake Tanganyika
Source: Biology (Basel). 2021 Aug 18;10(8):797. doi: 10.3390/biology10080797 (PMC8389663; doi:10.3390/biology10080797)
Supplement: Supplementary file 1 [file biology-10-00797-s001.zip › biology-1279428-supplementary.pdf]

Table S1: Measurements of *Cichlidogyrus casuarinus* from Mpulungu. Zambia collected from *Hemibates stenosoma* and *Bathybates graueri*. respectively (a – mean value±standard deviation. b – range).

Parameters (µm)	*C. casuarinus* ex *H. stenosoma*	*C. casuarinus* ex *B. graueri*
**Ventral anchor**		
**Total length**	52.3[a]±2.1 (48.5 – 52.3)[b]; n= 37	50.1 (46.7 – 51.7); n=4
**Length to notch**	43.8±2.4 (38.8 – 47.4); n=37	42.9 (40.8 – 47.2); n=4
**Inner root length**	15.4±2.8 (11.2 – 23.3); n=37	42.9 (40.8 – 47.2); n=4
**Outer root length**	7.8±2.0 (4.7 – 12.1); n=36	9.8 (5.4 – 12.1); n=4
**Point length**	14.4±2.0 (11.9 – 16.6); n=36	15.1 (13.7 – 17.6); n=4
**Dorsal anchor**		
**Total length**	58.0±2.1 (53.8 – 62.2); n=34	59.5 (55.3 – 63.5); n=4
**Length to notch**	41.0±3.1 (32.4 – 48.5); n=34	42.0 (38.8 – 44.8); n=4
**Inner root length**	21.8±3.1 (15.0 – 26.1); n=35	22.2 (17.2 – 25.1); n=4
**Outer root length**	7.7±1.9 (4.0 – 11.7); n=34	8.2 (7.5 – 8.9); n=4
**Point length**	13.2±1.2 (10.6 – 15.6); n=33	12.2 (10.5 – 14.1); n=4
**Ventral bar**		
**Branch length**	63.5±7.1 (44.7 – 78.5); n=37	69.8 (65.3 – 73.6); n=4
**Branch maximum width**	8.6±1.2 (6.7 – 11.7); n=37	8.6 (7.6 – 9.6); n=4
**Dorsal bar**		
**Maximum straight width**	79.4±8.1 (64.8 – 98.4); n=36	89.4 (77.8 – 94.6); n=4
**Thickness at midlength**	14.0±2.1 (10.6 – 18.7); n=37	16.3 (14.0 – 17.8); n=4
**Distance between auricles**	29.7±5.1 (18.0 – 38.9); n=37	37.1 (32.7 – 40.7); n=4
**Auricle length**	16.2±2.4 (12.6 – 22.6); n=38	18.9 (15.9 – 20.1); n=4
**Hooks**		
**Pair I**	33.6±3.0 (25.8 – 42.2); n=36	34.3 (31.9 – 35.5); n=4
**Pair II**	23.0±1.7 (19.5 – 26.7); n=33	24.2 (23.1 – 26.4); n=4
**Pair III**	23.8±1.7 (20.7 – 27.7); n=33	24.8 (24.4 – 25.6); n=4
**Pair IV**	25.0±1.9 (22.1 – 29.1); n=34	26.2 (22.8 – 28.1); n=4
**Pair V**	11.0±1.2 (9.0 – 13.0); n=22	10.6 (9.1 – 11.4); n=4
**Pair VI**	26.6±2.2 (22.1 – 31.1); n=36	27.1 (25.5 – 28.5); n=4
**Pair VII**	27.3±2.2 (23.6 – 32.6); n=32	30.1 (28.4 – 32.7); n=4
**Copulatory tube straight length**	35.2±1.8 (30.8 – 30.3); n=36	36.8 (32.1 – 39.9); n=5
**Accessory piece curved length**	34.1±5.3 (22.0 – 43.0); n=21	33.1 (31.0 – 36.7); n=4
**Heel straight length**	53.1±7.7 (37.7 – 74.1); n=36	74.1 (63.4 – 89.9); n=4

Table S2: Overview of the length (in bp) of rDNA regions in the partly assembled ribosomal operon of *Cichlidogyrus casuarinus* based on a pooled sample of 80 individuals (GenBank accession number MZ700354).

**Region**	**Position**	**Length**
SSU	1 – 1974	1974
ITS1	1975– 2457	482
5.8	2458 – 2614	156
ITS2	2615 – 3129	514
LSU	3130– 7014	3884

Table S3: Overview of the allele frequencies recovered from different datasets in the analysed fragment of *cox*1 gene (392 bp).

**Position**	**Individual-based sequences from Mpulungu, September 2018**				**Individual-based sequencing of *C. casuarinus* available**				**Pooled NGS dataset from Mpulungu, September 2018**			
	**A**	**T**	**C**	**G**	**A**	**T**	**C**	**G**	**A**	**T**	**C**	**G**
**1**	0.83333	0	0.16667	0	0.94118	0	0.05882	0	NA	NA	NA	NA
**2**	NA	NA	NA	NA	0	0.76471	0.23529	0	NA	NA	NA	NA
**5**	NA	NA	NA	NA	0.23529	0.76471	0	0	NA	NA	NA	NA
**11**	0	0.54167	0.375	0.08333	0	0.57353	0.39706	0.02941	0	0.03272	0.96728	0
**14**	0	0.58333	0.41667	0	0	0.58824	0.41176	0	NA	NA	NA	NA
**17**	0.20833	0.79167	0	0	0.10294	0.89706	0	0	0.04410	0.95590	0	0
**20**	0	0.41667	0.58333	0	0	0.39706	0.60294	0	0	0.45170	0.54830	0
**23**	0.95833	0	0.04167	0	0.98529	0	0.01471	0	NA	NA	NA	NA
**24**	0	0.95833	0.04167	0	0	0.98529	0.01471	0	NA	NA	NA	NA
**26**	0.37500	0	0	0.625	0.33824	0	0	0.66176	0.38401	0	0	0.61599
**32**	NA	NA	NA	NA	0.01471	0.95588	0.02941	0	0	0.95670	0.01083	0.03248
**41**	NA	NA	NA	NA	0	0.01471	0	0.98529	0	0.01060	0	0.98940
**47**	NA	NA	NA	NA	0	0.01471	0.98529	0	0	0.03748	0.96252	0
**50**	NA	NA	NA	NA	0	0.01471	0.98529	0	NA	NA	NA	NA
**62**	NA	NA	NA	NA	0	0.01471	0.98529	0	NA	NA	NA	NA
**68**	0.95833	0.04167	0	0	0.98529	0.01471	0	0	NA	NA	NA	NA
**74**	NA	NA	NA	NA	NA	NA	NA	NA	0	0.98780	0.01220	0
**80**	NA	NA	NA	NA	0.01471	0	0	0.98529	0.01648	0	0	0.98352
**92**	0	0.45833	0.54167	0	0	0.44118	0.55882	0	0	0.51558	0.48442	0
**93**	NA	NA	NA	NA	0	0.02941	0.97059	0	NA	NA	NA	NA
**98**	NA	NA	NA	NA	0	0.98529	0.01471	0	NA	NA	NA	NA
**102**	NA	NA	NA	NA	NA	NA	NA	NA	0.01801	0	0	0.98199
**110**	NA	NA	NA	NA	0	0.01471	0.98529	0	0	0.02710	0.97290	0
**114**	0	0.95833	0	0.04167	0	0	0.98529	0.01471	NA	NA	NA	NA

**118**	NA	NA	NA	NA	NA	NA	NA	NA	0	0.00270	0.99730	0
**119**	0.95833	0.04167	0	0	0.98529	0.01471	0	0	0.98111	0.01889	0	0
**122**	NA	NA	NA	NA	0	0.98529	0.01471	0	NA	NA	NA	NA
**128**	NA	NA	NA	NA	NA	NA	NA	NA	0	0.02929	0.97071	0
**129**	NA	NA	NA	NA	0	0.98529	0.01471	0	NA	NA	NA	NA
**131**	NA	NA	NA	NA	NA	NA	NA	NA	0.01887	0	0	0.98113
**134**	0	0.95833	0.041667	0	0	0.98529	0.01471	0	NA	NA	NA	NA
**148**	NA	NA	NA	NA	0.01471	0	0.98529	0	0.00265	0	0.99735	0
**150**	NA	NA	NA	NA	NA	NA	NA	NA	0.00262	0	0.99738	0
**152**	0	0.62500	0.375	0	0	0.63235	0.36765	0	0	0.54993	0.45007	0
**155**	0	0.95833	0.04167	0	0	0.94118	0.05882	0	0	0.91777	0.08223	0
**161**	NA	NA	NA	NA	NA	NA	NA	NA	0	0.99346	0.00654	0
**167**	NA	NA	NA	NA	0.98529	0	0	0.01471	NA	NA	NA	NA
**176**	NA	NA	NA	NA	0	0.98529	0.01471	0	0	0	0	0
**185**	NA	NA	NA	NA	NA	NA	NA	NA	0	0.98496	0.01504	0
**186**	NA	NA	NA	NA	0.97059	0	0.02941	0	NA	NA	NA	NA
**187**	NA	NA	NA	NA	0.01471	0	0.98529	0	NA	NA	NA	NA
**191**	NA	NA	NA	NA	0	0.98529	0.01471	0	NA	NA	NA	NA
**194**	0	0.95833	0.04167	0	0	0.98529	0.01471	0	NA	NA	NA	NA
**200**	0	0.79167	0.20833	0	0	0.26471	0.73529	0	0	0.22914	0.77086	0
**202**	NA	NA	NA	NA	0	0.01471	0.98529	0	NA	NA	NA	NA
**203**	0.625	0.375	0	0	0.67647	0.30882	0.01471	0	0.59141	0.40859	0	0
**206**	NA	NA	NA	NA	0.014706	0	0	0.98529	0	0.00240	0	0.99760
**218**	NA	NA	NA	NA	0	0.01471	0.98529	0	NA	NA	NA	NA
**233**	NA	NA	NA	NA	NA	NA	NA	NA	0.99269	0	0	0.00731
**254**	0.04167	0	0	0.95833	0.029412	0	0	0.97059	NA	NA	NA	NA
**257**	0	0.83333	0.16667	0	0	0.82353	0.17647	0	0	0.86510	0.13490	0
**265**	NA	NA	NA	NA	NA	NA	NA	NA	0	0.00247	0.99753	0

**266**	0.16667	0	0	0.83333	0.08824	0	0	0.91176	0.04074	0.01605	0	0.94321
**269**	NA	NA	NA	NA	0.02941	0.97059	0	0	NA	NA	NA	NA
**274**	NA	NA	NA	NA	0.98529	0.01471	0	0	NA	NA	NA	NA
**281**	NA	NA	NA	NA	NA	NA	NA	NA	0.98422	0	0	0.01578
**283**	NA	NA	NA	NA	NA	NA	NA	NA	0	0.00243	0	0.99757
**284**	0.95833	0	0	0.04167	0.97059	0	0	0.02941	0.97800	0	0	0.02200
**293**	NA	NA	NA	NA	NA	NA	NA	NA	0	0.02303	0	0.97697
**299**	NA	NA	NA	NA	NA	NA	NA	NA	0.01613	0	0	0.98387
**302**	0.91667	0	0	0.08333	0.95588	0	0	0.04412	0.96500	0	0	0.03500
**305**	NA	NA	NA	NA	0.01471	0	0	0.98529	NA	NA	NA	NA
**308**	NA	NA	NA	NA	NA	NA	NA	NA	0	0.98642	0.01358	0
**313**	NA	NA	NA	NA	NA	NA	NA	NA	0.00244	0	0.99756	0
**317**	0	0.95833	0.04167	0	0	0.073523	0.92647	0	0	0.05897	0.94103	0
**334**	NA	NA	NA	NA	NA	NA	NA	NA	0	0.99006	0	0.00994
**338**	0	0.95833	0.04167	0	0	0.95588	0.04412	0	0	0.97146	0.01117	0.01737
**342**	0	0.04167	0	0.95833	0	0.01471	0	0.98529	NA	NA	NA	NA
**344**	NA	NA	NA	NA	0	0.01471	0.98529	0	0	0.01363	0.98637	0
**347**	NA	NA	NA	NA	NA	NA	NA	NA	0.97767	0	0	0.02233
**348**	NA	NA	NA	NA	0	0.98529	0.01471	0	NA	NA	NA	NA
**350**	NA	NA	NA	NA	NA	NA	NA	NA	0	0.99009	0.00991	0
**356**	0.04167	0.04167	0	0.91667	0.01471	0.01471	0	0.97059	NA	NA	NA	NA
**357**	0	0.54167	0.45833	0	0	0.39706	0.60294	0	0	0.42428	0.57572	0
**359**	0.95833	0	0.041667	0	0.98529	0	0.01471	0	NA	NA	NA	NA
**362**	0	0.04167	0.95833	0	0	0.01471	0.98529	0	0.00503	0.01887	0.97610	0
**365**	0	0.04167	0.95833	0	0	0.02941	0.97059	0	0	0.01880	0.98120	0
**377**	0	0.41667	0.58333	0	0	0.41176	0.58824	0	0	0.42522	0.57478	0
**379**	0.04167	0	0	0.95833	0.01471	0	0	0.98529	NA	NA	NA	NA
**381**	NA	NA	NA	NA	NA	NA	NA	NA	0.00247	0	0.99753	0

**383**	NA	NA	NA	NA	0.01471	0.01471	0.97059	0	0	0.03453	0.96547	0
**383**	NA	NA	NA	NA	0	0.98529	0.01471	0	NA	NA	NA	NA
**386**	NA	NA	NA	NA	0	0.01471	0	0.98529	NA	NA	NA	NA
**389**	NA	NA	NA	NA	0	0.01471	0.98529	0	0	0.00498	0.99502	0
**392**	NA	NA	NA	NA	0	0.98529	0.01471	0	0	0.99499	0.00501	0

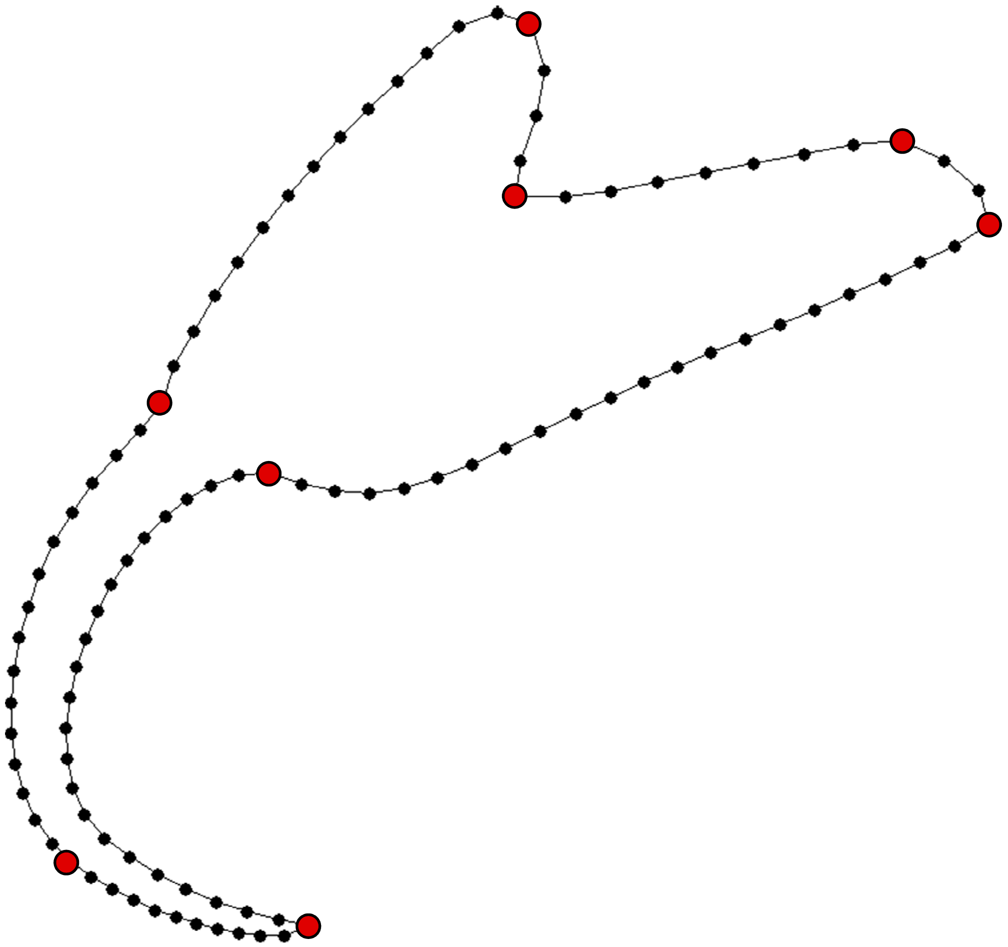

Fig. S1: Position of fixed landmarks (big points) as well as semi-landmarks (small points) on the dorsal anchor of *Cichlidogyrus casuarinus*. Number and position of landmarks was followed in the analyses of the ventral anchor.
